# Silver and Copper Nanoparticles Induce Oxidative Stress in Bacteria and Mammalian Cells

**DOI:** 10.3390/nano12142402

**Published:** 2022-07-14

**Authors:** Thelma Ameh, Matthew Gibb, Dinny Stevens, Sahar H. Pradhan, Evan Braswell, Christie M. Sayes

**Affiliations:** 1 Department of Environmental Science, Baylor University, Waco, TX 76798, USA; thelma_ameh1@baylor.edu (T.A.); dinny_stevens1@baylor.edu (D.S.); sahar_pradhan@baylor.edu (S.H.P.); 2 Institute of Biomedical Studies, Baylor University, Waco, TX 76798, USA; matthew_gibb@baylor.edu; 3 Mission Laboratory, United States Department of Agriculture, Animal and Plant Health Inspection Service, Plant Protection and Quarantine, Science and Technology, Edinburg, TX 78541, USA; evan.braswell@usda.gov

**Keywords:** cetyltrimethylammonium bromide, polyvinyl pyrrolidone, reactive oxygen species, metal ion, *E. coli*, BEAS-2B

## Abstract

Silver and copper nanoparticles (AgNPs and CuNPs) coated with stabilizing moieties induce oxidative stress in both bacteria and mammalian cells. Effective antibacterial agents that can overcome existing mechanisms of antibacterial resistance will greatly improve biomedical interventions. In this study, we analyzed the effect of nanoparticle-induced stress. *Escherichia coli* and normal human bronchial epithelial (BEAS-2B) cells were selected for this study. The nanoparticle constructs tested showed low toxicity to mammalian cells except for the polyvinylpyrrolidone-surface-stabilized copper nanoparticles. In fact, both types of copper nanoparticles used in this study induced higher levels of reactive oxygen species than the surface-stabilized silver nanoparticles. In contrast to mammalian cells, the surface-stabilized silver and copper nanoparticles showed varying levels of toxicity to bacteria cells. These data are expected to aid in bridging the knowledge gap in differential toxicities of silver and copper nanoparticles against bacteria and mammalian cells and will also improve infection interventions.

## 1. Introduction

Effective antibacterial agents that can overcome existing mechanisms of antibacterial resistance will greatly improve biomedical interventions [1,2]. To address this challenge, metal-based nanomaterials are extensively studied in terms of antibacterial properties. For example, silver nanoparticles (AgNPs) and copper nanoparticles (CuNPs) have been coated with polymers to improve particle stability and have shown toxicities in both bacteria and mammalian cells [3,4,5,6,7,8,9]. However, the modes of toxicological action by which these nanoparticles induce stress are speculative, and have not been clearly demonstrated. There are three main working hypotheses. First, AgNPs and CuNPs induce toxicity through metal-ion leaching resulting in soluble metal ions that can directly interact with cells and induce cytotoxicity [10]. Second, these nanoparticles induce toxicity through reactive-oxygen-species (ROS) generation and subsequent oxidative stress [11]. Third, non-oxidative mechanisms, such as cell membrane permeability disruption can induce cell death [12]. Toxic modes of action from nanoparticle exposure in different cell types can occur simultaneously or independent of one another, but most exposure scenarios lead to cell death [13,14]. Therefore, it is important to characterize the specific modes of nanoparticle toxicity in different cell types.

Metal ions that leach from nanoparticles can serve as effective antibacterial agents capable of combating antibiotic resistance because the presumed mode of action is significantly different from that of conventional antibiotics (which target cell wall assembly, protein synthesis and DNA replication) [15]. Non-essential metal ions like silver induce toxicity to organisms even at low concentrations, while essential metal ions such as copper, iron and zinc are important for cellular metabolism and are needed for normal cellular functions [16,17]. However, even essential metals can become toxic when the intracellular concentrations rise above usable biological thresholds [18]. The caveat of applying metal ions for biomedical applications involves optimizing properties at extremely low concentrations that impact positive effects on cells without causing death.

A property of metal nanoparticles that has received some attention in antibacterial-agent research is the effect of particle surface coating (and conferred surface charge). Polydopamine (PDA) coating was found to significantly increase the antimicrobial efficiency of AgNPs against *Escherichia coli*, via catechol-rich PDA–Ag chemical interactions which increased generation of ROS and led to significant damage to the bacterial cell membrane [19]. Other studies have observed polyvinyl-pyrrolidone (PVP) stabilizing agent to promote the growth of *Escherichia coli* and *Sphingobacterium multivorum,* showing more viable bacteria colonies than no-treatment control groups against these bacteria [20,21]. This observation could be explained with the assumption that PVP acts as a growth-promoting factor for bacteria. It has been shown that the high water-binding capacity of PVP aids the metabolism of cells by maintaining moisture content in the cell media and that PVP binds to bacteria toxins released into media during the stationary growth phase, thereby extending the time before they reach the death phase.

Surface charge of metal nanoparticles has also been shown to influence antibacterial efficacy. The negatively charged bacterial cell membrane resulting from the presence of amino, carboxyl and phosphate groups has a higher tendency to react closely with positively charged nanoparticle surfaces via electrostatic attraction, thereby inducing membrane leakage and nanoparticle entry into cells where they can impair bacteria growth [22,23]. Conversely, electrostatic repulsion occurs when bacteria are exposed to negatively charged nanoparticle surfaces, thus limiting chemical interactions at the nanoparticle–cell interface [24]. The antibacterial activity of charged AgNPs on the growth of *Staphylococcus aureus*, *Streptococcus mutans*, *Streptococcus pyogenes*, *Escherichia coli* and *Proteus vulgaris* was found to be most effective for positively charged AgNPs and least effective for negatively charged AgNPs, while the neutrally charged particles showed intermediate antibacterial efficacy [24]. Taken together, there is strong evidence in the scientific literature stating that nanoparticle-surface-stabilizing agents which confer a charge to the nanoparticle surface induce bacterial cell death.

Several mechanisms have been proposed for the antibacterial action of metal nanoparticles. The commonly cited mechanisms are intracellular toxicity of leached metal ions and induced oxidative stress via increased ROS production [25,26]. In this study, we analyzed the effect of nanoparticle-induced oxidative stress in both bacteria and mammalian cells in a dose-dependent manner. *Escherichia coli* K-12 strain MG1655 (ATCC 700926, *E. coli* K-12), a non-pathogenic strain, was selected for this study and has been extensively used to study the impact of nanoparticle exposures on bacteria cells [27,28]. Normal human bronchial epithelial (BEAS-2B) cells were selected for this study and have been extensively used in nanotoxicological studies to evaluate the effects of pulmonary exposure to nanoparticles [29,30,31]. By performing a study that uses the same metal nanoparticles at the same concentrations on two different biological models, a systematic comparison can be drawn to inform research projects moving forward.

## 2. Materials and Methods

**Figure 1:**
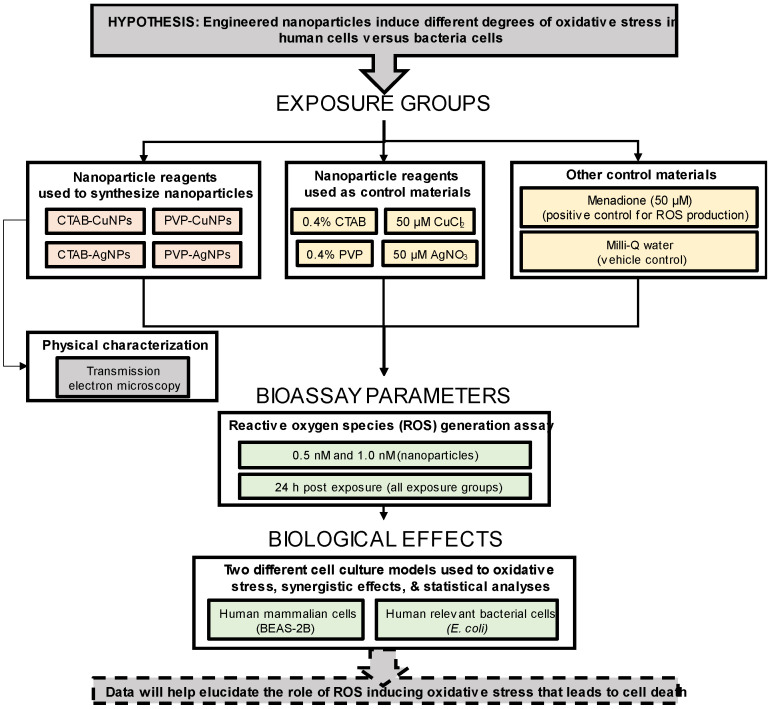
Experimental design showing exposure variables to determine induced reactive-oxygen-species (ROS) generation in bacteria and mammalian cells after nanoparticle exposure.

Experimental design. The major objective of this study was to assess the extent to which four unique monodisperse-engineered nanoparticles could produce reactive oxygen species and subsequently induce oxidative stress in two different cell culture models. The general approach to this study was to systematically produce, characterize and test the chemical properties (e.g., surface functional groups), physical properties (e.g., size and shape) and biological properties (e.g., induced cytotoxicity and growth inhibition) of the aqueous suspended nanoparticles using the Turkevich method of chemical reduction and seeded growth. Figure 1 outlines the remaining strategy. The reagents used to produce the nanoparticles (i.e., CTAB, PVP, CuCl_2_ and AgNO_3_) served as control materials for this experiment, menadione served as a positive ROS-generating control and Milli-Q water served as a negative control.

Reagents. All chemicals used in the study were acquired at the highest purity available. Silver nitrate (AgNO_3_, CAS# 7761-88-8, > 99.9%) was purchased from Ricca Chemical Company (Arlington, TX, USA). Polyvinylpyrrolidone (PVP, CAS# 9003-39-8, MW 40,000), sodium hydroxide (NaOH, CAS# 1310-73) and copper (II) nitrate trihydrate (Cu(NO_3_)_2_·3H_2_O, CAS# 10031-43-3) were used. Cetyltrimethylammonium bromide (CTAB, CAS# 57-09-0) was purchased from Bio-world (Dublin, OH, USA). Glucose (C_6_H_12_O_6_, CAS# 50-99-7), L-ascorbic acid (C_6_H_8_O_6_, CAS# 50-81-7), copper (II) chloride dihydrate (CuCl_2_.2H_2_O, CAS#10125-13-0) and hydrazine hydrate (H_4_N_2_.H_2_O, CAS# 10217-52-4) were purchased from Acros Organics (Thermo Fisher Scientific, Waltham, MA, USA). ROS-Glo™ H_2_O_2_ Assay was purchased from Promega (Southampton, UK). Dulbecco’s modified Eagle’s medium (DMEM)/F12, fetal bovine serum (FBS), 0.05% trypsin-EDTA enzyme, Gibco™ Penicillin-Streptomycin and Remel nutrient broth were purchased from Thermo Fisher Scientific (Waltham, MA, USA). Human bronchial epithelial cell line (BEAS-2B cells) and *Escherichia coli* K-12 strain MG1655 (ATCC 700926, *E. coli* K-12) were purchased from the American Type Culture Collection (ATCC) (Manassas, VA, USA).

Nanoparticle Synthesis. Briefly, the following nanoparticles were prepared and used in this study. Polyvinyl-pyrrolidone-stabilized silver nanoparticles (termed PVP-AgNPs) were synthesized in a one-step process in which 2 mL of freshly prepared 5% silver nitrate (AgNO_3_) solution was added dropwise into 100 mL of 2% PVP solution and left to react under vigorous stirring at 100 °C for 1 h [32,33]. For the synthesis of cetyltrimethylammonium-bromide-stabilized silver nanoparticles (termed CTAB-AgNPs), 50 mL of freshly prepared 0.01 M AgNO_3_ solution was added dropwise into a reaction vessel containing 50 mL of 0.01 M CTAB under vigorous magnetic stirring [34]. Then, 50 mL of 0.01 M sodium hydroxide (NaOH) was immediately added to 25 mL of 5.0 mM glucose (C_6_H_12_O_6_) solution. The AgNO_3_-CTAB complex was then added to the NaOH-glucose solution and left to react for 5 h at 50 °C under vigorous magnetic stirring until nanoparticle formation was complete.

The synthesis of PVP-stabilized copper nanoparticles (termed PVP-CuNPs) was performed with a 1:1 mixture of 0.8 M PVP and 0.4 M L-ascorbic acid (C_6_H_8_O_6_) solution to a 1:1 mixture 0.8 M PVP and 0.01 M copper (II) nitrate trihydrate Cu(NO_3_)_2_·3H_2_O. The mixture was then left in a reaction vessel under vigorous magnetic stirring at 45 °C until nanoparticle formation was complete after a 3 h reaction time [35]. Copper nanoparticles stabilized with CTAB (termed CTAB-CuNPs) were synthesized by adding a 1:1 mixture of 0.01 M CTAB and 0.08 M hydrazine hydrate (H_6_N_2_O) to a 1:1 mixture of 0.01 M CTAB and 1.0 mM copper (II) chloride (CuCl_2_.2H_2_O). The pH was adjusted to 10 and the reaction proceeded under vigorous magnetic stirring for 3 h. For purification, all nanoparticle suspensions were centrifuged at 8000 rpm for 30 min and the supernatant was stored at ambient temperatures. Pellets were re-suspended in a known volume of ultrapure water prior to characterization. Moreover, confirmation of all particles synthesized was performed through UV-Vis spectroscopy.

Nanoparticle Imaging. Morphological characterization of the nanoparticles was performed using transmission electron microscopy (TEM, JEOL JEM-1010 TEM, Tokyo, Japan). Carbon-coated copper grids (Electron Microscopy Sciences; Hartfield, PA, USA) were covered with a drop of nanoparticle suspension, dried under a hot plate and viewed on the TEM at an accelerating voltage of 60 kV to obtain electron micrographs of the nanoparticles.

Mammalian Cell Culture. BEAS-2B cells, an adherent epithelial cell line derived from a human bronchus, were maintained in Dulbecco’s modified Eagle’s medium (DMEM)/F12 (Catalog No. 11-320-082; Fisher Scientific, Hampton, VA, USA) media supplemented with 10% heat-inactivated fetal bovine serum (FBS; Catalog No. 10-082-147; Fisher Scientific, Hampton, VA, USA) and 1% penicillin-streptomycin (Catalog No. MT300002CI; Fisher Scientific, Hampton, VA, USA). Cells were grown and maintained in a 75 cm^2^ cell culture flask at 37 °C in a 5% CO_2_ humidified incubator. Culture medium was changed every 2 days and microscopically inspected for changes indicating bacterial infection. For seeding, 0.05% trypsin-EDTA enzyme was added to cells on reaching 80–90% confluence. Trypsinized cells were added to equal parts media and centrifuged at 300× *g* for 5 min, supernatant removed, resuspended in 1 mL complete media and 10 µL of cell suspension was added to trypan blue dye in 1:1 ratio and counted on a Countess 3 FL automated cell counter (Invitrogen, Waltham, MA, USA).

Bacteria Cell Culture. *Escherichia coli* K-12 strain MG1655 (ATCC 700926, *E. coli* K-12), a non-pathogenic strain, was used for all bacterial experiments. Exponentially grown bacteria cells were obtained by inoculating overnight colonies of *E. coli* cell suspension in Remel nutrient broth and incubated at 37 °C for 6 h in an orbital shaker at 100 rpm. Optical density (OD) was measured at 600 nm prior to plating cells.

Measurement of Reactive Oxygen Species. To measure ROS generation, the luminescent assay ROS-Glo™ H_2_O_2_ Assay was used to analyze oxidative stress 24 h post exposure. This kit has previously been utilized in a variety of lung cell types [36,37]. Mammalian cells were plated in flat-bottom clear, white polystyrene (TC)-treated 96-well plates (MilliporeSigma, Burlington, MA, USA) to reach recommended confluence of 0.04 × 10^6^ cells per well. Bacterial cells were serially diluted to log phase of 1 × 10^6^ colony forming units (CFU) and plated in the same well plates as mammalian cells. The assay was performed according to the manufacturer’s instructions, including menadione as positive control at 50 µM for mammalian cells and 200 µM for bacterial cells. Menadione is a known inducer of cellular ROS production and was used as a confirmed indicator of ROS production in cells. Final luminescence of samples was measured using the Synergy H1 microplate reader (BioTek, Winooski, VT, USA).

Experiments were performed using four different nanoparticle constructs (CTAB-CuNPs, CTAB-AgNPs, PVP-CuNPs, PVP-AgNPs) at 0.5 and 1.0 µM concentrations against BEAS-2B mammalian cells and *E. coli* bacteria cells. In addition, 5 different control materials were also tested; these include the positive control menadione, CuCl_2_, AgNO_3_, CTAB and PVP. Silver nitrate and copper chloride were used to show evidence of metal ion poisoning and toxicity while the stabilizing agents were used to determine if the presence of stabilizing agents on the nanoparticle surface influence observed ROS production after nanoparticle exposure to cells.

Measurement of Nanoparticle Chemical Interactions. It is not known (or there is no general consensus) whether the inhibitory effects of nanoparticles are due to the metal core or the stabilizing agent. Here, the fractional inhibitory concentration equation has been adapted to calculate the interaction index of the nanoparticle constructs used in this study [38]. To determine the relationship between stabilizing agent and metal core in induced ROS response, the interaction index is taken as the ratio of measured ROS value for each surface-stabilized metal nanoparticle to the predicted ROS value, which is the combined sum of calculated ROS values of the silver nitrate or copper chloride and the stabilizing agent. If the effects are purely additive, the interaction index is expected to equal 1, while values less than 1 indicate an antagonistic relationship, where one component suppresses the inhibitory effect of the other, and values greater than 1 signify a synergistic relationship, where one component enhances the inhibitory effect of the other.

Statistical Analysis. Statistical significance of ROS production was obtained from one way analysis of variance (ANOVA) measurements. A Student *t*-test was performed to verify statistical significance of interaction indices. All statistical tests were performed in JMP^©^, Version 13 (SAS Institute Inc, Cary, NC, USA, 2016).

## 3. Results

**Figure 2:**
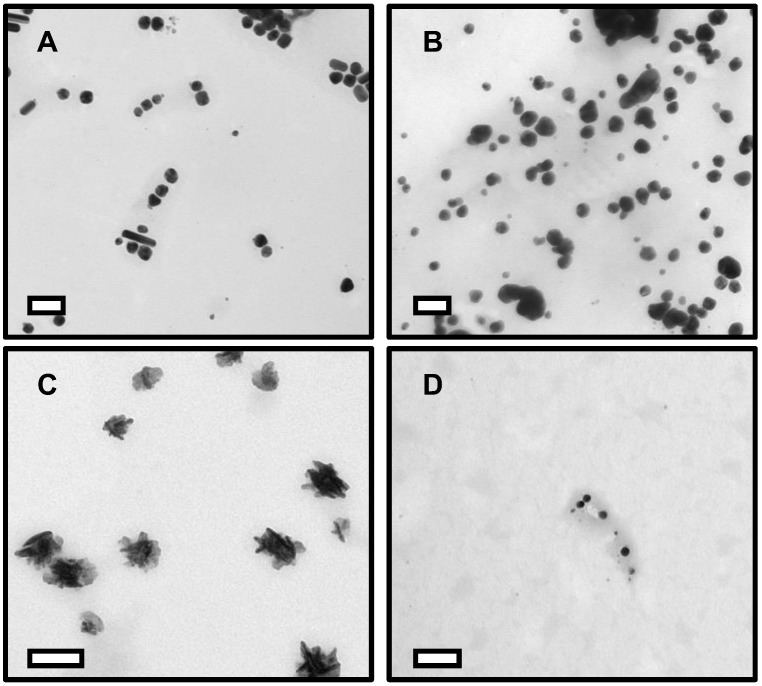
Transmission electron microscopy (TEM) images of surface-stabilized silver and copper nanoparticles. (**A**) CTAB-AgNPs (**B**) PVP-AgNPs (**C**) CTAB-CuNPs (**D**) PVP-CuNPs. Scale bar represents 100 nm.

Figure 2 shows transmission electron microscopy (TEM) images of the surface-stabilized silver and copper nanoparticles. The CTAB-AgNPs are 59 ± 15 nm in diameter with +18.2 mV zeta potential value, (Figure 2A) vary in shape from rough spheres to rod-like particles and exhibited moderate particle aggregation. PVP-AgNPs are 75 ± 20 nm in diameter with −5.84 mV zeta potential value, show low aggregation and are roughly spherical in shape (Figure 2B). CTAB-CuNPs are also roughly spheroidal in shape with low particle aggregation and a size of 89 ± 11 nm in diameter with +18.6 mV zeta potential value (Figure 2C), while PVP-CuNPs are 22 ± 5 nm in diameter with −0.57 mV zeta potential value and spherical in shape with low aggregation (Figure 2D).

**Figure 3:**
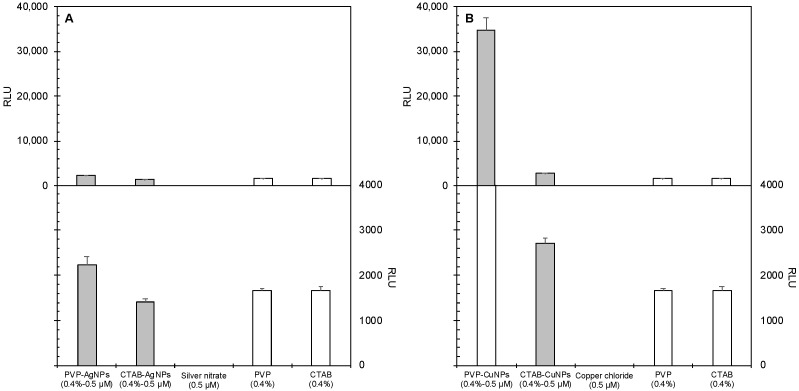
Effects of nanoparticle systems on ROS generation by BEAS-2B mammalian cells treated with two exposure concentrations, namely 0.5 µM (data shown) and 1.0 µM (data not shown). The top panels (**A**,**B**) show the same data as the bottom panels, respectively, but at different scales. The top panel shows that the PVP-CuNP is drastically larger than the others, while the bottom panel allows comparison between the smaller values. (**A**) Relative luminescence units (RLU) versus silver treatment type. (**B**) RLU versus copper treatment type. Error bars depict one standard deviation from the mean. The assay was validated using menadione as a positive control for ROS production in mammalian cells; its RLU value was 84,500.

The effects of nanoparticle exposure to BEAS-2B mammalian cells are shown in Figure 3. Figure 3A shows ROS production in BEAS-2B cells after exposure to 0.5 µM and 1.0 µM concentrations of the surface-stabilized silver and copper nanoparticles. Figure 3B shows ROS production after exposure to the control materials. Nanoparticles produced relatively lower levels of ROS in BEAS-2B mammalian cells at both concentrations tested except for PVP-stabilized copper nanoparticles (PVP-CuNPs), which produced statistically significant ROS (*p* < 0.0001). Surface-stabilized silver nanoparticles did not show elevated ROS levels regardless of stabilizing agent.

**Figure 4:**
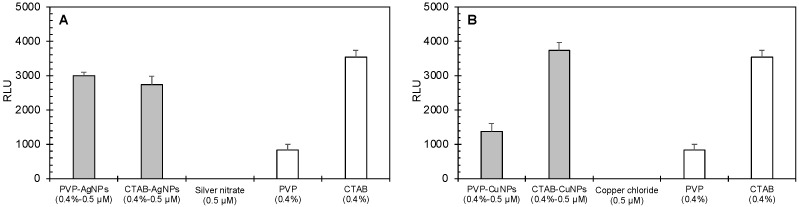
Effects of nanoparticle systems on ROS generation by *E. coli* bacteria cells exposed to surface-coated nanoparticles and their component chemicals. Panels show the relative luminescence units resulting from the ROS-Glo™ H_2_O_2_ Assay of E. coli cells exposed to (**A**) surface-coated silver nanoparticles and their components and (**B**) surface-coated copper nanoparticles and their components. Error bars depict one standard deviation from the mean. The assay was validated using menadione as a positive control for ROS production in bacterial cells; its RLU value was 3650.

The effects of nanoparticle exposure to the bacteria cell type, *E. coli,* are shown in Figure 4. Figure 4A shows ROS production in *E. coli* after exposure to 0.5 µM and 1.0 µM concentrations of the surface-stabilized silver and copper nanoparticles. Figure 4B shows ROS production after exposure to the control materials.

While the PVP-CuNPs produced the highest amount of ROS in mammalian cell culture, these particles produced the least amount of ROS in cultures of *E. coli* bacteria cells at both concentrations tested. Among the suite of nanoparticles tested, CTAB-CuNPs produced the most ROS in *E. coli* at both experimental concentrations. Surface-stabilizing agents also produced ROS, with CTAB generating more ROS than PVP. ROS production by CTAB-CuNPs at both concentrations tested and 0.4% CTAB were statistically significant from all other exposure scenarios (*p* < 0.0001). This implies that ROS production in bacteria cells is primarily driven by the presence of CTAB in the CTAB-stabilized NPs. AgNO_3_ and CuCl_2_ also produced only slightly significant amounts of ROS (*p* < 0.0001) more than 0.5 nM PVP-CuNPs and 0.4% PVP.

**Figure 5:**
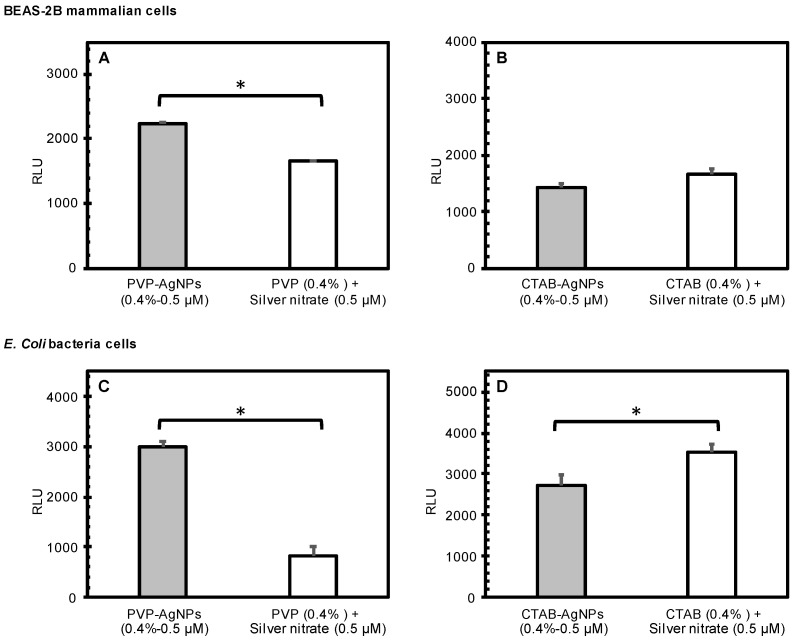
Comparison of the effects of surface-stabilized silver nanoparticles (measured) to the predicted cumulative effects of stabilizing agents and silver nitrate as calculated from independent component treatments. The graphs show the average RLU generated by treatment of BEAS-2B mammalian cells (**A**,**B**) and *E. coli* bacterial cells (**C**,**D**) with PVP-AgNPs (0.5 µM; (**A**,**C**)) and CTAB-AgNPs (0.5 µM; (**B**,**D**)). Error bars depict one standard deviation from the mean. Significantly different means are indicated by an asterisk (*p* < 0.05).

**Figure 6:**
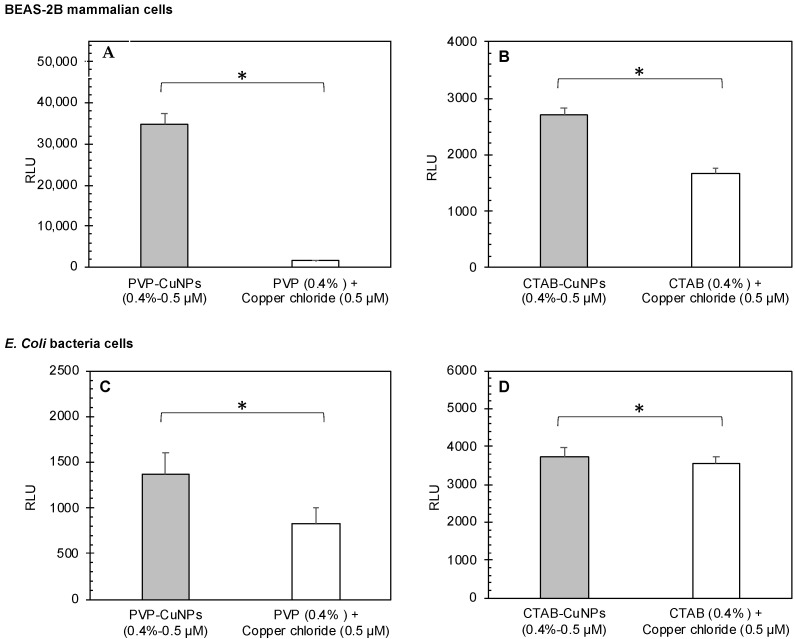
Calculated (effects of stabilizing agents plus effects of copper chloride) versus measured (surface-stabilized copper nanoparticle) relative luminescence units (RLU). The different copper nanoparticles (0.5 and 1.0 µM PVP-CuNPs and 0.5 and 1.0 µM CTAB-CuNPs) against (**A**,**B**) BEAS-2B mammalian cells and (**C**,**D**) *E. coli* bacterial cells. In each panel, the measured RLU is presented as the average of triplicate measurements, and the error bar shows the standard deviation; the asterisk indicates that the measured RLU was significantly lower than the calculated RFU (*p <* 0.05), indicating a consistent synergistic effect for PVP-CuNPs in both mammalian and bacterial cells (except for 0.5 nM PVP-CuNPs, where the results are equivalent) and contradicting effects for CTAB-CuNPs in mammalian cells (antagonistic effect) versus bacterial cells (synergistic effect). Black asterisks indicate that the surface-stabilized copper nanoparticle is significantly higher in RLUs than the 0.4% stabilizing agent plus 50 µM copper chloride co-exposure. Red asterisks indicate that the surface-stabilized copper nanoparticle is significantly lower in RLUs than the 0.4% stabilizing agent plus 50 µM copper chloride co-exposure.

Nanoparticle dose did not show significant differences in induced ROS production in both cell types. Figure 5 reports the effects of the AgNPs, while Figure 6 reports the effects of CuNPs. Generally, the surface-stabilized copper nanoparticles (CuNPs) induced higher levels of ROS production than the surface stabilized silver nanoparticles (AgNPs) in both mammalian and bacteria cells. PVP-AgNPs induced higher levels of ROS production than CTAB-AgNPs in both mammalian and bacteria cells. Overall, CTAB-CuNPs caused the highest amount of ROS production in bacteria cells while PVP-CuNPs caused the highest amount of ROS production in mammalian cells.

Table 1 summarizes the synergistic analysis of surface-stabilized AgNPs and CuNPs on induced ROS generation in mammalian and bacteria cells. Apart from CTAB-AgNP effects in mammalian cells, the silver nanoparticles showed chemical interactions responsible for induced ROS production in both mammalian and bacteria cells. This indicates that the interaction of metal-core composition and surface-stabilizing agent are responsible for the observed toxicological response of ROS induction. The same observation was also made for the surface-stabilized copper nanoparticles. There was an additive relationship between metal-ion effect and stabilizing-agent effect for PVP-CuNPs at the 0.5 µM concentration.

## 4. Discussion

The nanoparticles tested showed low toxicity to mammalian cells except for PVP-CuNPs, and the surface-stabilized copper nanoparticles also generally induced higher levels of ROS than surface-stabilized silver nanoparticles. The stabilizing agents also did not produce significant levels of ROS. Toxicity due to excessive metal-ion exposure seems to be the main driver of induced ROS production in mammalian cells with silver nitrate and copper chloride producing more ROS than stabilizing agents. In particular, CuCl_2_ produced more ROS than AgNO_3_ and this would explain the slightly higher difference in ROS produced by the surface-stabilized copper nanoparticles than the silver nanoparticles. Overall, the nanoparticles that produced the most reactive oxygen species were PVP-CuNPs.

In contrast to mammalian cells, the surface stabilized silver and copper nanoparticles all showed varying levels of toxicity to bacteria cells at the concentrations tested, as evidenced by ROS generation. CTAB-CuNPs produced the most ROS in *E. coli* at both experimental concentrations. Surface-stabilizing agents also produced ROS, with CTAB generating more ROS than PVP. ROS production in bacteria cells is primarily driven by the presence of CTAB in the CTAB-stabilized metal NPs and to a lesser extent by the metal-core composition of the nanoparticle construct. Induced ROS production in bacteria cells after exposure to surface-stabilized metal nanoparticles is driven mainly by nanoparticle effects, as indicated by chemical interactions of the nanoparticle constituents and to a lesser extent by metal-ion poisoning.

Role of oxidative stress. Release of metal ions has been shown to induce the generation of ROS in bacteria and mammalian cells. Intracellular ROS is produced as a defense mechanism by cells and is cleared by antioxidants to maintain homeostasis. Production of ROS becomes detrimental to cells when the concentration being produced exceeds the antioxidant clearing process and this leads to damage of cellular macromolecules such as DNA, proteins and lipids. Ultimately, accumulation of ROS causes oxidative stress, leading to cell death [39,40,41]. Cao et al. found that a novel silver-Bi_2_MoO_6_ nanocomposite exhibits outstanding antibacterial activity through enhanced ROS production due to the chemodynamic and photodynamic synergistic capabilities between silver and Bi_2_MoO_6_ [41].

Metal-induced ROS production in bacteria cells can occur via three major routes. The first pathway is via Fenton and Fenton-like reactions of metals such as Fe, Cu, Cr and Ni, which create hydroxyl radicals from hydrogen peroxide using iron (II) and these other metal ions as catalyst [42,43,44]. Disrupted cellular function of iron-binding ligands is another pathway to metal-ROS-induced bacteria cell death; aluminum (Al), Cu and Ag have been shown to disrupt electron transfer reactions by targeting ferredoxins (iron–sulfur proteins), resulting in uncontrolled release of Fe into the cytoplasm and subsequent increased ROS generation [45]. Depletion of cellular antioxidant reservoirs has also been implicated as a pathway to metal-induced ROS production, as seen by the depletion of glutathione through its oxidization by thiophilic metals like Ag(I), cadmium (Cd)(II) or As (III) [16,46].

Role of metal-ion leaching. Mechanisms of cellular toxicity due to metal ion poisoning have been attributed to the affinity of metal ions for intracellular components and biomolecules and possibly to the resultant formation of metal–biomolecule complexes which have downstream effects on cellular function [47]. Cellular toxicity of metal ions can occur via different pathways: binding/blocking functional groups of biomolecules, displacing essential metals required for the catalytic activity of enzymes, binding to cellular thiol pool, thereby limiting antioxidant defenses to oxidative stress and participating in harmful chemical reactions in the cell [48,49,50,51,52]. Binding of metals to cellular thiols has been shown to have several consequences such as depleted antioxidant levels resulting in altered intracellular redox balance and ROS formation, increased vulnerability of proteins to attack by metals and ROS as well as impaired rate of repair mechanisms for oxidized thiols [48]. Metal-ion poisoning ultimately leads to deleterious effects to the cell such as damage to biomolecules such as proteins, lipids and nucleic acids, disruption and destruction of biological membranes, altered enzyme activity and cellular physiology processes and increased oxidative stress [47,49].

Cell membrane permeability disruption. The bacteria cell envelope is the point of entry into the cell and has membrane proteins and lipids that can interact with metal nanoparticles. Binding of metal nanoparticles to the phospholipid bilayer disrupts membrane permeability by reducing the dipole potential of the membrane, leading to nanoparticle entry into the cells and resultant ROS formation [53,54,55,56]. Chemical interaction of metal nanoparticles with the cell membrane can also result in lipid peroxidation, which damages cell membrane and increase cytoplasmic leakage [57,58]. Copper has been shown to cause lipid peroxidation in bacteria cell membranes and is implicated as a mechanism of the copper-mediated antibacterial effect [59].

## 5. Conclusions

The global persistence of antimicrobial resistance and the slow rate of development for new antimicrobial agents necessitates the need to explore non-traditional methods of antimicrobial therapy, hence the burgeoning research efforts on the use of metal-based nanoparticles as potential antimicrobial agents. Preclinical and clinical studies are important to explore the pharmacokinetic and safety implications of metal nanoparticles as alternative antibacterial agents; this is critical to accelerating their use in combatting active bacterial infections. The potential cytotoxicity of metal nanoparticles to mammalian and bacterial cells was explored in this study. Results showed that exposure to CTAB-AgNPs, CTAB-CuNPs and PVP-AgNPs induced low oxidative stress to mammalian cells, evidenced by low levels of generated ROS, while bacterial cells were more susceptible to cell damage after these specific metal nanoparticle exposures, as observed by elevated levels of ROS. Increased application of metal nanoparticles in the industrial, consumer and healthcare sectors poses a key challenge in unknown and unintended consequences to human and environmental health. Understanding the mechanistic pathways of antibacterial toxicity of metal nanoparticles will aid in bridging the knowledge gap and will also improve the development of new strategies of antibacterial therapy while simultaneously addressing questions about human toxicity and safety implications.

## Figures and Tables

**Table 1:** Summary of chemical-interaction analyses of nanoparticle components. ROS generation, measured by relative luminescence units (RLU), by mammalian and bacterial cells after exposure to surface-coated nanoparticles (Measured) or their components (Predicted). Measured and predicted RLU values and interaction indices against mammalian (BEAS-2B) and bacteria (*E. coli*) cells of each nanoparticle used in this study as compared to the individual constituents of the nanoparticle construct. Four types of nanoparticle constructs were tested, PVP-AgNPs, CTAB-AgNPs, PVP-CuNPs, and CTAB-CuNPs. Each RLU value was recorded from the assay measuring the reactive oxygen species. The table includes the analyses of effects after inoculation with 0.5 µM (only); the data collected using the 1.0 µM concentration produced similar results in terms of RLU values, interactions indices and statistical significance. The measured RLU value was significantly higher for PVP-AgNPs, PVP-CuNPs and CTAB-CuNPs tested in mammalian cells. The measured RLU values were significantly higher for PVP-AgNPs and PVP-CuNPs tested in bacteria cells.

Cell Type	Concentration ^a^	Measured RLU	Predicted RLU	Interaction Index $\left(\frac{Measured}{Predicted}\right)$		Statistical Significance
		Mean ± StDev	Mean ± StDev			*t*-Test ^b^
Silver Nanoparticles						
Mammalian	0.5 µM PVP-AgNPs	2250 ± 163	1660 ± 51.6	**1.35**	Synergism	4.18 × 10^−3^ *
	0.5 µM CTAB-AgNPs	1420 ± 77.4	1670 ± 86.9	**0.85**	Antagonism	1.85 × 10^−2^ *
Bacteria	0.5 µM PVP-AgNPs	3000 ± 104	834 ± 163	**3.60**	Synergism	3.23 × 10^−5^ *
	0.5 µM CTAB-AgNPs	2730 ± 250	3540 ± 205	**0.77**	Antagonism	0.159
**Copper Nanoparticles**						
Mammalian	0.5 µM PVP-CuNPs	34,800 ± 2680	1660 ± 51.6	**20.9**	Synergism	2.82 × 10^−5^ *
	0.5 µM CTAB-CuNPs	2700 ± 116	1670 ± 86.9	**1.62**	Synergism	2.49 × 10^−4^ *
Bacteria	0.5 µM PVP-CuNPs	1370 ± 230	834 ± 163	**1.64**	Synergism	3.05 × 10^−3^ *
	0.5 µM CTAB-CuNPs	3740 ± 235	3540 ± 205	**1.06**	Additivity	2.82 × 10^−2^ *

^a^ Concentration of surface-stabilized nanoparticles; ^b^ Statistical significance from Student’s *t*-test using two samples assuming equal variances; * Indicates statistical significance (*p* < 0.05).

## Data Availability

Not applicable.

## References

[1] Douafer H., Andrieu V., Phanstiel O., Brunel J.M. (2019). Antibiotic adjuvants: Make antibiotics great again!. J. Med. Chem..

[2] Laws M., Shaaban A., Rahman K.M. (2019). Antibiotic resistance breakers: Current approaches and future directions. FEMS Microbiol. Rev..

[3] Djoković V., Krsmanović R., Božanić D.K., McPherson M., Van Tendeloo G., Nair P.S., Georges M.K., Radhakrishnan T. (2009). Adsorption of sulfur onto a surface of silver nanoparticles stabilized with sago starch biopolymer. Colloids Surf. B Biointerfaces.

[4] Wahab M.A., Li L., Li H., Abdala A. (2021). Silver nanoparticle-based nanocomposites for combating infectious pathogens: Recent advances and future prospects. Nanomaterials.

[5] Dong B., Belkhair S., Zaarour M., Fisher L., Verran J., Tosheva L., Retoux R., Gilson J.P., Mintova S. (2014). Silver confined within zeolite EMT nanoparticles: Preparation and antibacterial properties. Nanoscale.

[6] Wahab M.A., Hasan C.M., Alothman Z.A., Hossain M.S.A. (2021). In-situ incorporation of highly dispersed silver nanoparticles in nanoporous carbon nitride for the enhancement of antibacterial activities. J. Hazard. Mater..

[7] Jaiswal S., Bhattacharya K., McHale P., Duffy B. (2015). Dual effects of β-cyclodextrin-stabilised silver nanoparticles: Enhanced biofilm inhibition and reduced cytotoxicity. J. Mater. Sci. Mater. Med..

[8] Greulich C., Braun D., Peetsch A., Diendorf J., Siebers B., Epple M., Köller M. (2012). The toxic effect of silver ions and silver nanoparticles towards bacteria and human cells occurs in the same concentration range. RSC Adv..

[9] Ismail N.A., Shameli K., Wong M.M.T., Teow S.Y., Chew J., Sukri S.N.A.M. (2019). Antibacterial and cytotoxic effect of honey mediated copper nanoparticles synthesized using ultrasonic assistance. Mater. Sci. Eng. C.

[10] Nagy A., Harrison A., Sabbani S., Munson R.S., Dutta P.K., Waldman W.J. (2011). Silver nanoparticles embedded in zeolite membranes: Release of silver ions and mechanism of antibacterial action. Int. J. Nanomed..

[11] Gurunathan S., Han J.W., Dayem A.A., Eppakayala V., Kim J.-H. (2012). Oxidative stress-mediated antibacterial activity of graphene oxide and reduced graphene oxide in Pseudomonas aeruginosa. Int. J. Nanomed..

[12] Leung Y.H., Ng A.M., Xu X., Shen Z., Gethings L.A., Wong M.T., Chan C.M., Guo M.Y., Ng Y.H., Djurišić A.B. (2014). Mechanisms of antibacterial activity of MgO: Non-ROS mediated toxicity of MgO nanoparticles towards Escherichia coli. Small.

[13] Akter M., Sikder M.T., Rahman M.M., Ullah A.A., Hossain K.F.B., Banik S., Hosokawa T., Saito T., Kurasaki M. (2018). A systematic review on silver nanoparticles-induced cytotoxicity: Physicochemical properties and perspectives. J. Adv. Res..

[14] Fröhlich E.E., Fröhlich E. (2016). Cytotoxicity of nanoparticles contained in food on intestinal cells and the gut microbiota. Int. J. Mol. Sci..

[15] Liu Y., Imlay J.A. (2013). Cell death from antibiotics without the involvement of reactive oxygen species. Science.

[16] Lemire J., Harrison J., Turner R. (2013). Box 3: The Fenton reaction, free radical chemistry and metal poisoning. Nat. Rev. Microbiol..

[17] Soren S., Kumar S., Mishra S., Jena P.K., Verma S.K., Parhi P. (2018). Evaluation of antibacterial and antioxidant potential of the zinc oxide nanoparticles synthesized by aqueous and polyol method. Microb. Pathog..

[18] Crisponi G., Nurchi V.M. (2011). Metal ion toxicity. Encyclopedia of Inorganic and Bioinorganic Chemistry.

[19] Niyonshuti I.I., Krishnamurthi V.R., Okyere D., Song L., Benamara M., Tong X., Wang Y., Chen J. (2020). Polydopamine surface coating synergizes the antimicrobial activity of silver nanoparticles. ACS Appl. Mater. Interfaces.

[20] Biradar B., Santhosh G. (2018). Role of polymeric additives in formulation, shelf-life and bioefficacy of liquid inoculant of Pseudomonas fluoresens. Int. J. Pure Appl. Biosci..

[21] Deaker R., Roughley R.J., Kennedy I.R. (2004). Legume seed inoculation technology—A review. Soil Biol. Biochem..

[22] Mahlapuu M., Håkansson J., Ringstad L., Björn C. (2016). Antimicrobial Peptides: An Emerging Category of Therapeutic Agents. Front. Cell. Infect. Microbiol..

[23] Van der Wal A., Norde W., Zehnder A.J., Lyklema J. (1997). Determination of the total charge in the cell walls of Gram-positive bacteria. Colloids Surf. B Biointerfaces.

[24] Abbaszadegan A., Ghahramani Y., Gholami A., Hemmateenejad B., Dorostkar S., Nabavizadeh M., Sharghi H. (2015). The effect of charge at the surface of silver nanoparticles on antimicrobial activity against gram-positive and gram-negative bacteria: A preliminary study. J. Nanomater..

[25] Hang M.N., Gunsolus I.L., Wayland H., Melby E.S., Mensch A.C., Hurley K.R., Pedersen J.A., Haynes C.L., Hamers R.J. (2016). Impact of nanoscale lithium nickel manganese cobalt oxide (NMC) on the bacterium Shewanella oneidensis MR-1. Chem. Mater..

[26] Imlay J.A. (2013). The molecular mechanisms and physiological consequences of oxidative stress: Lessons from a model bacterium. Nat. Rev. Microbiol..

[27] Graves J.L., Tajkarimi M., Cunningham Q., Campbell A., Nonga H., Harrison S.H., Barrick J.E. (2015). Rapid evolution of silver nanoparticle resistance in Escherichia coli. Front. Genet..

[28] McQuillan J.S., Shaw A.M. (2014). Differential gene regulation in the Ag nanoparticle and Ag_+_-induced silver stress response in Escherichia coli: A full transcriptomic profile. Nanotoxicology.

[29] Abbas I., Badran G., Verdin A., Ledoux F., Roumie M., Guidice J.M.L., Courcot D., Garçon G. (2019). In vitro evaluation of organic extractable matter from ambient PM2. 5 using human bronchial epithelial BEAS-2B cells: Cytotoxicity, oxidative stress, pro-inflammatory response, genotoxicity, and cell cycle deregulation. Environ. Res..

[30] Zheng L., Liu S., Zhuang G., Xu J., Liu Q., Zhang X., Deng C., Guo Z., Zhao W., Liu T. (2017). Signal transductions of BEAS-2B cells in response to carcinogenic PM2. 5 exposure based on a microfluidic system. Anal. Chem..

[31] Wu J., Shi Y., Asweto C.O., Feng L., Yang X., Zhang Y., Hu H., Duan J., Sun Z. (2017). Fine particle matters induce DNA damage and G2/M cell cycle arrest in human bronchial epithelial BEAS-2B cells. Environ. Sci. Pollut. Res..

[32] Samadi N., Hosseini S., Fazeli A., Fazeli M. (2010). Synthesis and antimicrobial effects of silver nanoparticles produced by chemical reduction method. DARU J. Pharm. Sci..

[33] Wang H., Qiao X., Chen J., Wang X., Ding S. (2005). Mechanisms of PVP in the preparation of silver nanoparticles. Mater. Chem. Phys..

[34] Khan Z., Al-Thabaiti S.A., El-Mossalamy E., Obaid A.Y. (2009). Studies on the kinetics of growth of silver nanoparticles in different surfactant solutions. Colloids Surf. B Biointerfaces.

[35] Wu C., Mosher B.P., Zeng T. (2006). One-step green route to narrowly dispersed copper nanocrystals. J. Nanoparticle Res..

[36] Scheffler S., Dieken H., Krischenowski O., Förster C., Branscheid D., Aufderheide M. (2015). Evaluation of E-cigarette liquid vapor and mainstream cigarette smoke after direct exposure of primary human bronchial epithelial cells. Int. J. Environ. Res. Public Health.

[37] Bathrinarayanan P.V., Brown J.E., Marshall L.J., Leslie L.J. (2018). An investigation into E-cigarette cytotoxicity in-vitro using a novel 3D differentiated co-culture model of human airways. Toxicol. Vitr..

[38] Rakholiya K.D., Kaneria M.J., Chanda S.V. (2013). Medicinal plants as alternative sources of therapeutics against multidrug-resistant pathogenic microorganisms based on their antimicrobial potential and synergistic properties. Fighting Multidrug Resistance with Herbal Extracts, Essential Oils and Their Components.

[39] De Lucca Camargo L., Touyz R.M. (2019). Textbook of Vascular Medicine.

[40] Wang E., Huang Y., Du Q., Sun Y. (2017). Silver nanoparticle induced toxicity to human sperm by increasing ROS (reactive oxygen species) production and DNA damage. Environ. Toxicol. Pharmacol..

[41] Cao C., Zhang T., Yang N., Niu X., Zhou Z., Wang J., Yang D., Chen P., Zhong L., Dong X. (2022). POD Nanozyme optimized by charge separation engineering for light/pH activated bacteria catalytic/photodynamic therapy. Signal Transduct. Target. Ther..

[42] Warnes S.L., Keevil C.W. (2016). Lack of involvement of Fenton chemistry in death of methicillin-resistant and methicillin-sensitive strains of Staphylococcus aureus and destruction of their genomes on wet or dry copper alloy surfaces. Appl. Environ. Microbiol..

[43] Laha D., Pramanik A., Maity J., Mukherjee A., Pramanik P., Laskar A., Karmakar P. (2014). Interplay between autophagy and apoptosis mediated by copper oxide nanoparticles in human breast cancer cells MCF7. Biochim. Biophys. Acta (BBA)-Gen. Subj..

[44] Müller K., Skepper J.N., Posfai M., Trivedi R., Howarth S., Corot C., Lancelot E., Thompson P.W., Brown A.P., Gillard J.H. (2007). Effect of ultrasmall superparamagnetic iron oxide nanoparticles (Ferumoxtran-10) on human monocyte-macrophages in vitro. Biomaterials.

[45] Mustila H., Allahverdiyeva Y., Isojärvi J., Aro E., Eisenhut M. (2014). The bacterial-type [4Fe–4S] ferredoxin 7 has a regulatory function under photooxidative stress conditions in the cyanobacterium Synechocystis sp. PCC 6803. Biochim. Biophys. Acta (BBA)-Bioenerg..

[46] Harrison J.J., Tremaroli V., Stan M.A., Chan C.S., Vacchi-Suzzi C., Heyne B.J., Parsek M.R., Ceri H., Turner R.J. (2009). Chromosomal antioxidant genes have metal ion-specific roles as determinants of bacterial metal tolerance. Environ. Microbiol..

[47] Hobman J.L., Crossman L.C. (2015). Bacterial antimicrobial metal ion resistance. J. Med. Microbiol..

[48] Meriga B., Reddy B.K., Rao K.R., Reddy L.A., Kishor P.K. (2004). Aluminium-induced production of oxygen radicals, lipid peroxidation and DNA damage in seedlings of rice (Oryza sativa). J. Plant Physiol..

[49] Hobman J.L., Yamamoto K., Oshima T. (2007). Molecular Microbiology of Heavy Metals.

[50] Alhasawi A., Auger C., Appanna V., Chahma M., Appanna V. (2014). Zinc toxicity and ATP production in Pseudomonas fluorescens. J. Appl. Microbiol..

[51] Xu F.F., Imlay J.A. (2012). Silver (I), mercury (II), cadmium (II), and zinc (II) target exposed enzymic iron-sulfur clusters when they toxify Escherichia coli. Appl. Environ. Microbiol..

[52] Chillappagari S., Seubert A., Trip H., Kuipers O.P., Marahiel M.A., Miethke M. (2010). Copper stress affects iron homeostasis by destabilizing iron-sulfur cluster formation in Bacillus subtilis. J. Bacteriol..

[53] Ortiz-Benítez E.A., Velázquez-Guadarrama N., Durán Figueroa N.V., Quezada H., Olivares-Trejo J.d.J. (2019). Antibacterial mechanism of gold nanoparticles on Streptococcus pneumoniae. Metallomics.

[54] Bondarenko O., Juganson K., Ivask A., Kasemets K., Mortimer M., Kahru A. (2013). Toxicity of Ag, CuO and ZnO nanoparticles to selected environmentally relevant test organisms and mammalian cells in vitro: A critical review. Arch. Toxicol..

[55] Calvano C.D., Picca R.A., Bonerba E., Tantillo G., Cioffi N., Palmisano F. (2016). MALDI-TOF mass spectrometry analysis of proteins and lipids in Escherichia coli exposed to copper ions and nanoparticles. J. Mass Spectrom..

[56] Brayner R., Ferrari-Iliou R., Brivois N., Djediat S., Benedetti M.F., Fiévet F. (2006). Toxicological impact studies based on Escherichia coli bacteria in ultrafine ZnO nanoparticles colloidal medium. Nano Lett..

[57] Wang L., Hu C., Shao L. (2007). The antimicrobial activity of nanoparticles: Present situation and prospects for the future. Int. J. Nanomed..

[58] Tiwari V., Mishra N., Gadani K., Solanki P.S., Shah N.A., Tiwari M. (2018). Mechanism of anti-bacterial activity of zinc oxide nanoparticle against carbapenem-resistant Acinetobacter baumannii. Front. Microbiol..

[59] Hong R., Kang T.Y., Michels C.A., Gadura N. (2012). Membrane lipid peroxidation in copper alloy-mediated contact killing of Escherichia coli. Appl. Environ. Microbiol..

